# Mast Cells Are Abundant in Primary Cutaneous T-Cell Lymphomas: Results from a Computer-Aided Quantitative Immunohistological Study

**DOI:** 10.1371/journal.pone.0163661

**Published:** 2016-11-28

**Authors:** Johanna Eder, Radu Rogojanu, Waltraud Jerney, Friedrich Erhart, Alexander Dohnal, Melitta Kitzwögerer, Georg Steiner, Julia Moser, Franz Trautinger

**Affiliations:** 1 Department of Dermatology and Venereology, Karl Landsteiner University of Health Sciences, St. Pölten, Austria; 2 Karl Landsteiner Institute of Dermatological Research, St. Pölten, Austria; 3 Department of Pathophysiology and Allergy Research, Medical University of Vienna, Vienna, Austria; 4 TissueGnostics GmbH, Vienna, Austria; 5 Department of Dermatology, Medical University of Vienna, Vienna, Austria; 6 Laboratory for Tumor Immunology, CCRI St. Anna Kinderkrebsforschung, Vienna, Austria; 7 Institute of Clinical Pathology, Karl Landsteiner University of Health Sciences, St. Pölten, Austria; Universita degli Studi di Bari Aldo Moro, ITALY

## Abstract

**Background:**

Mast cells (MC) are bone marrow derived haematopoetic cells playing a crucial role not only in immune response but also in the tumor microenvironment with protumorigenic and antitumorigenic functions. The role of MC in primary cutaneous T-cell lymphomas (CTCL), a heterogeneous group of non-Hodgkin lymphomas with initial presentation in the skin, is largely unknown.

**Objective:**

To gain more accurate information about presence, number, distribution and state of activation (degranulated vs. non-degranulated) of MC in CTCL variants and clinical stages.

**Materials and Methods:**

We established a novel computer-aided tissue analysis method on digitized skin sections. Immunohistochemistry with an anti-MC tryptase antibody was performed on 34 biopsies of different CTCL subtypes and on control skin samples. An algorithm for the automatic detection of the epidermis and of cell density based CTCL areas was developed. Cells were stratified as being within the CTCL infiltrate, in P1 (a surrounding area 0–30 μm away from CTCL), or in P2 (30–60 μm away from CTCL) area.

**Results:**

We found high MC counts within CTCL infiltrates and P1 and a decreased MC number in the surrounding dermis P2. Higher MC numbers were found in MF compared to all other CTCL subgroups. Regarding different stages of MF, we found significantly higher mast cell counts in stages IA and IB than in stages IIA and IIB. Regarding MC densities, we found a higher density of MC in MF compared to all other CTCL subgroups. More MC were non-degranulated than degranulated.

**Conclusion:**

Here for the first time an automated method for MC analysis on tissue sections and its use in CTCL is described. Eliminating error from investigator bias, the method allows for precise cell identification and counting. Our results provide new insights on MC distribution in CTCL reappraising their role in the pathophysiology of CTCL.

## Introduction

Among the characteristics of cancer is its ability to recruit normal infiltrating and resident cells to generate a specific microenvironment fostering malignant growth.[1]

One of these bystanders is the mast cell (MC) originally discovered by Paul Ehrlich more than 100 years ago and mainly known for its immunological effector function. MC are haematopoetic cells which leave the bone marrow as undifferentiated precursors finding final differentiation in their target tissues under the influence of several microenvironmental growth factors, in particular stem cell factor (SCF), the ligand for the c-kit receptor tyrosine kinase (CD117).[2–6]

Already in 1891 Eugen Westphal, a student of Ehrlich’s, recognized that MC populate the interface between developing tumors and healthy tissues.[7,8]

Since then, MC have been found to accumulate around and within many types of solid cancer. While in parasitic infections and allergies, MC have been investigated for decades, studies on MC in malignant tissues have been somewhat neglected since Westphal and Ehrlich’s discovery. Recent research has revealed inconsistent results, showing a positive as well as a negative relationship between the presence and number of MC and prognosis in human malignancies.[3]

Potential MC effects on tumor growth can be categorized as either direct effects on tumor cells, such as MC cytotoxicity, or as indirect effects, such as immune cell recruitment, tissue remodelling of the neighboring environment and MC-directed angiogenesis.[4]

Angiogenesis is stimulated through release of preformed pro-angiogenic factors from MC granules as vascular endothelial growth factor (VEGF), fibroblastic growth factor-2 (FGF-2), and also through serine proteases as tryptase and chymase.[2,9,10]

Tryptase promotes the proliferation of endothelial cells, vascular tube formation and also dissolves extracellular matrix to provide space for neovascular growth.[2]

Increased numbers of MC have been shown to correlate with tumor progression and poor prognosis in various human malignancies including carcinomas of breast, stomach, rectum, liver, bile ducts, pancreas, prostate, and lung.[11–18]

At the same time other studies found a correlation of high MC counts with good prognosis and improved patient survival in carcinomas of colon, breast, and ovaries and in non-small cell lung cancer.[19–25]

In renal cell cancer patients no correlation between MC numbers and prognosis was found.[26]

Similarly, inconsistent observations have been made in lymphoid neoplasms, with increased numbers of MC correlating with poor prognosis in Hodgkin lymphoma and B-cell non-Hodgkin lymphoma and with favorable outcome in diffuse large B-cell lymphomas.[27–30]

Primary cutaneous lymphomas (PCL) are defined as a diverse group of non-Hodgkin lymphomas (NHL) with primary presentation in the skin and no sign of extracutaneous disease at the time of diagnosis.[30]

The presence and role of MC in PCL is largely unknown. The skin is a common site of extranodal non-Hodgkin lymphoma (NHL), manifestation ranking only behind the gastrointestinal tract.[30]

According to European and US studies, the annual incidence of PCL is estimated to be 1:100,000 with primary cutaneous T-cell lymphomas (CTCL) counting for about 71–77% and primary cutaneous B-cell lymphomas for about 23–29% of PCL.[30,31]

The heterogeneous group of CTCL includes indolent variants, following a chronic course with slow progression over years and decades (e.g. mycosis fungoides (MF) and the CD30+ lymphoproliferative disorders lymphomatoid papulosis (LyP) and primary cutaneous anaplastic large cell lymphoma (C-ALCL)), and aggressive variants (e.g. Sezary syndrome (SS) and extranodal natural killer T-cell lymphoma, nasal type).[30,31]

Although the high density of MC in human skin together with their well known interaction with T-cells provide a rationale for the investigation of MC in CTCL, up to now only one study on MC in primary cutaneous lymphomas has been published demonstrating a protumorigenic role of MC in primary cutanous B- and T-cell lymphomas.[32]

In this and the other studies mentioned above MC numbers were estimated by microscopic counting of several high-power fields by one or more pathologists. The aim of our study was to gain more accurate information about presence, number and distribution of MC in CTCL by establishing and applying a novel computer-aided tissue analysis algorithm.

## Materials and Methods

Archival formalin-fixed, paraffin-embedded tissue samples were retrieved from 34 patients with different CTCL variants of which the majority were patients with different stages of MF (for patient characteristics see Table 1).

**Table 1:** Demographic data of 34 primary cutaneous T-cell lymphoma cases.

Type of lymphoma	n	Sex distribution male/female	Age distribution median	(years) range
CTCL	34	24/10	60,5	(13–85)
Mycosis fungoides	25	20/5	59	(13–85)
MF IA	16	14/2	61	(13–82)
MF IB	1	0/1	65	
MF IIA	1	1/0	54	
MF IIB	7	5/2	69	(23–85)
Sezary syndrome	2	0/2	n/a	(68,85)
Lymphomatoid papulosis	3	2/1	62	(53–79)
Primary cutaneous CD4^+^ small/medium pleomorphic T-cell lymphoma	3	2/1	58	(51–72)
CD30^+^ primary cutaneous anaplastic large-cell lymphoma	1	0/1	28	

n = number of patients; m = male, f = female; CTCL = primary cutaneous T-cell lymphoma; MF = Mycosis fungoides.

Only archival samples and historical data were analyzed anonymously precluding informed consent. All experiments were done in accordance with the local regulations and requirements regarding research on archival human tissues.

CTCL diagnosis was made according to the WHO-EORTC classification for cutaneous lymphomas.[30]

**Figs 1a and b:**
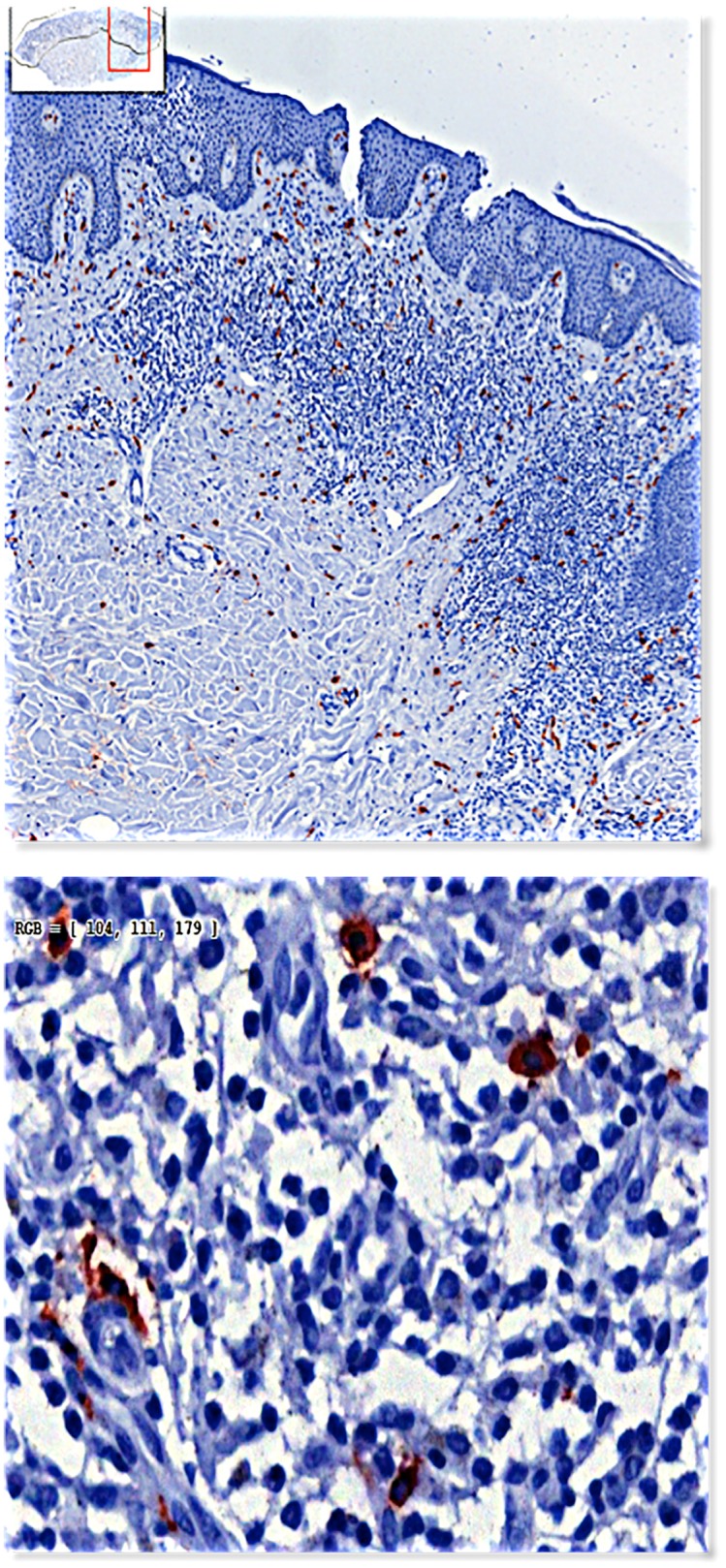
Immunohistochemistry staining with a monoclonal anti-mast cell tryptase antibody.

As controls we used biopsies from patients with inflammatory skin diseases (lichen planus, psoriasis, eczema) and normal skin. On all these biopsies we performed immunohistochemistry (Dako REAL Detection Systems, Dako, Denmark) utilizing a mouse monoclonal anti-mast cell tryptase antibody (Anti-Mast Cell Tryptase antibody, AA1, prediluted, ab74506, Abcam, Cambridge, UK) and appropriate alkaline phosphatase labeled second step reagents (Dako) resulting in a combination with 3-amino-9-ethylcarbazole (AEC) as substrate in MC stained in red. Counterstaining was done with hematoxylin (Fig 1a and 1b).

Skin sections were scanned using TissueFAXS 4.2 and analyzed using StrataQuest 5.0 (both TissueGnostics Gmbh, Vienna, Austria). Images were acquired with 20x magnification at 0.275 μm/pixel and further analysed by adaptive guided segmentation (AGS) as described previously.[33,34]

**Figs 2a–c:**
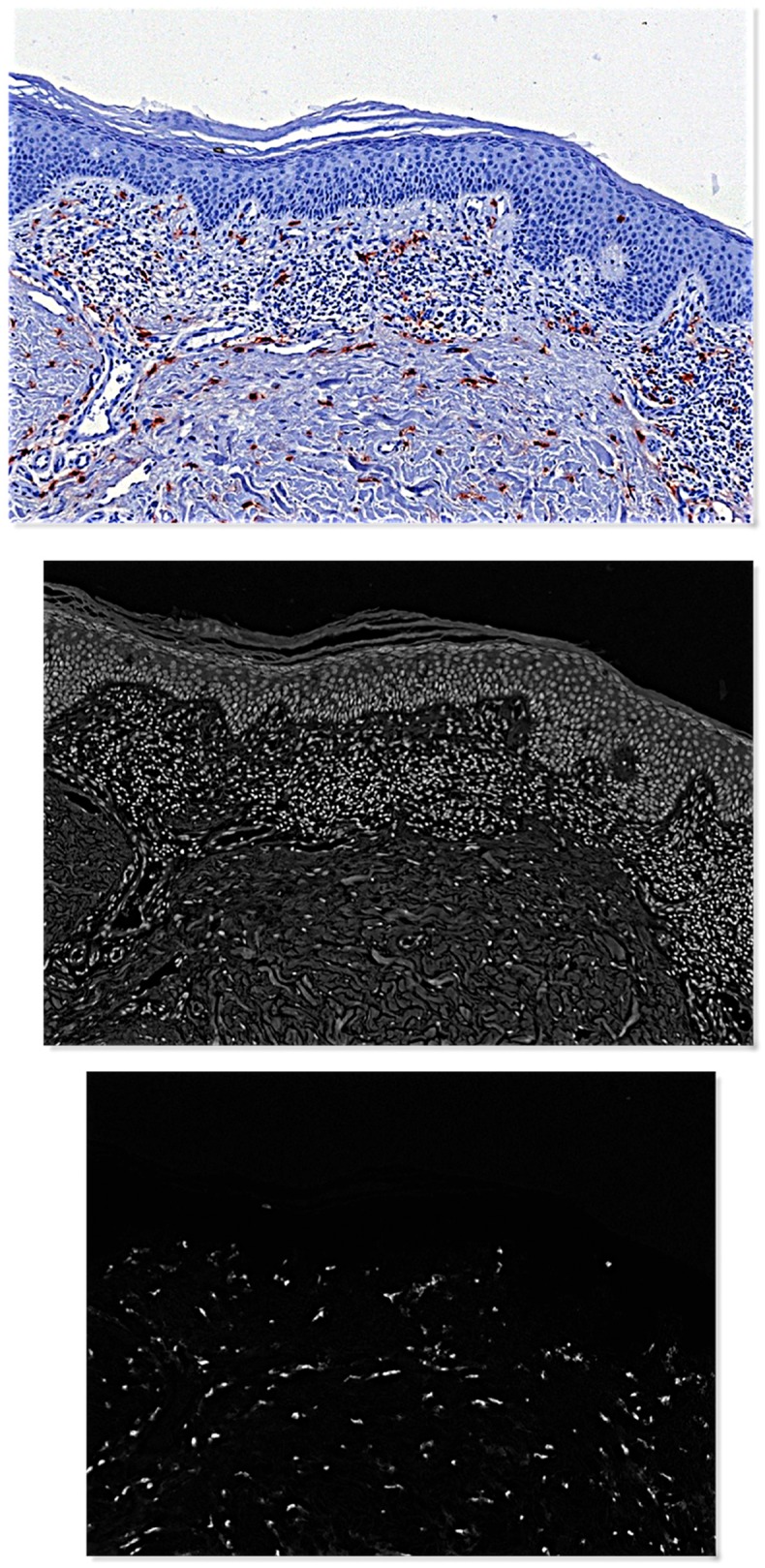
Color deconvolution to seperate the hematoxylin stained nuclei from tryptase positive mast cells.

The same principles were applied to extract dermis and epidermis masks after color deconvolution to generate hematoxylin (H) and tryptase (T) optical density images (Fig 2a, 2b and 2c). Within the dermis masks, cells were detected by nuclear segmentation applied on H images. Local cell densities were estimated using the Parzen window method.[34]

**Fig 3:**
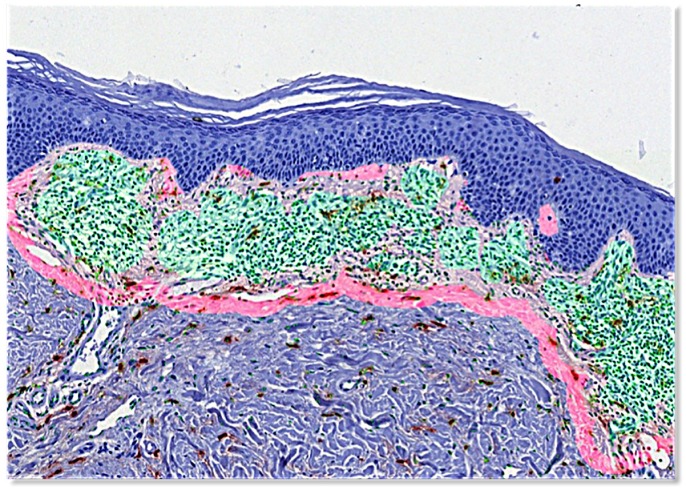
Regions of interest. Lymphoma infiltrate: green. P1 (0 to 30 micron from lymphoma infiltrate): light pink. P2 (30 and 60 micron from lymphoma infiltrate): pink.

Through another AGS step CTCL areas were extracted automatically, visually corrected as needed, and by distance transformation two proximity areas (P1 = 0 to 30 μm and P2 = 30 to 60 μm outside CTCL areas) were defined (Fig 3). MC were identified on T images by local clustering. Single large compact blobs were defined as non-degranulated MC, while clusters with fragmented blobs were counted as degranulated MC (S1 Fig). Finally, cell counts and densities of all cells, all MC, degranulated and non-degranulated MC were extracted for each of the defined areas (CTCL, P1 and P2).

### Statistical analysis

Absolute and relative MC densities are given as MC/mm^2^ and as percent of total cells in CTCL areas, respectively. Degranulated MC are given as percent of all MC. Given the limited sample size, non-parametric methods were applied for statistical testing. For descriptive statistics median and range were calculated. For paired group comparisons the Wilcoxon matched-pairs signed rank test was used where appropriate and for unpaired group comparisons the Mann-Whitney U test was used. Both are non-parametric tests. P values of less than 0.05 were considered significant.

## Results

### MC counts are increased in the area immediately surrounding CTCL infiltrates and in early stages compared to later stages of MF

Analysis of MC distribution in the predefined regions (CTCL area, P1, P2) revealed that MC numbers were identical in P1 (median 229.66 MC/mm^2^; range 24.27–543.21 MC/mm^2^) and CTCL areas and dropped significantly from P1 to P2 (median 169.20 MC/mm^2^; range 13.09–444.71 MC/mm^2^; p = 0.001)

Comparing the number and distribution of mast cells in CTCL areas (n = 34, median 219.55 MC/mm^2^; range 10.28–447.63 MC/mm^2^) and in dermis from normal donors (n = 3, median 114.06 MC/mm^2^; range 97.52–147.82 MC/mm^2^) we found a non-significant trend towards higher MC counts in CTCL (p = 0,248). Conversely, higher MC counts were found in samples of inflammatory skin diseases (median 391,27 MC/mm^2^; range 361,28–1332,45 MC/mm^2)^ than in CTCL (see Table 2). Common to all variants of CTCL represented by more than one case is the wide range of MC counts reducing the statistical power to detect significant differences in limited samples.

**Table 2:** Mast cell counts per mm^2^ in different CTCL subtypes.

Type of lymphoma			MC/mm^2^ median (range)		
	n	Tumor	P1	P2	Dermis
**CTCL**	34	220 (10–448)	230 (24–543)	169 (13–445)	
**Mycosis fungoides**	25	281 (53–448)	242 (56–543)	192 (43–445)	
**MF IA**	16	308 (53–448)	543	185 (69–319)	
**MF IB**	1	402	421	445	
**MF IIA**	1	291	189 (56–377)	391	
**MF IIB**	17	107 (61–337)	(44,261)	137 (43–284)	
**Sezary syndrome**	2	(10,338)	122 (52–274)	(13,209)	
**LyP**	3	135 (44–241)	181 (24–203)	99 (16–221)	
**SM**	3	104 (25–168)	31	125 (16–160)	
**C-ALCL**	1	35		25	
**Normal Skin**	3				114 (98–148)
**ISD**	3				391 (361–1332)

MC = mast cells; CTCL = primary cutaneous T-cell lyphomas; MF = Mycosis fungoides; LyP = lymphomatoid papulosis; SM = primary cutaneous CD4^+^ small/medium sized pleomorphic T-cell lymphoma; C-ALCL = primary cutaneous anaplastic large cell lymhpoma; ISD = inflammatory skin diseases.

**Fig 4:**
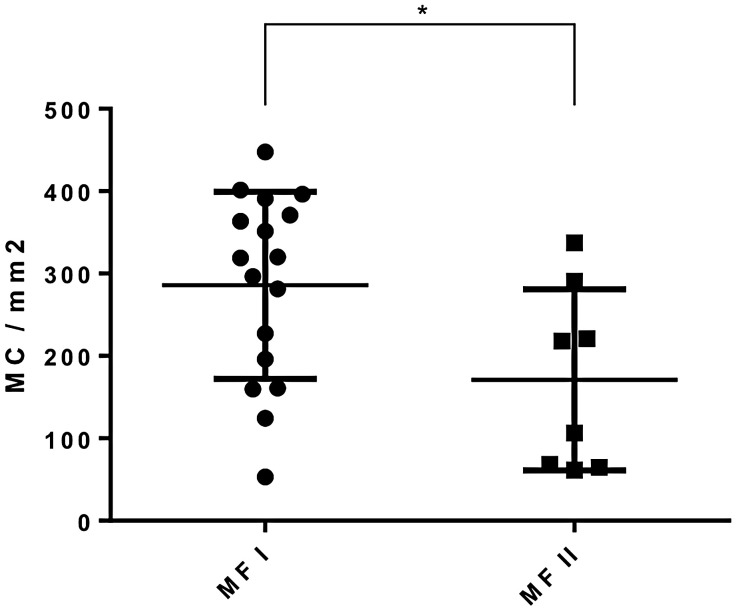
Mast cell count per mm^2^ as measured by tissueFAXS system showing the difference between combined early MF stages IA and IB and combined advanced stages MF IIA and IIB. Significantly higher mast cell counts were found in stage I disease.

Looking at different stages of MF we found significantly higher mast cell counts in the CTCL areas in combined early stages IA and IB (median 354.58 MC/ mm^2^; range 53.1–447.63 MC/mm^2^, n = 17) than in combined advanced stages IIA and IIB (median 198.52 MC/mm^2^; range 61.39–337.09 MC/mm^2^, n = 18, p = 0.031) (see Fig 4).

### Relative MC density is increased in MF compared to all other CTCL variants

We were not only interested in MC counts/mm^2^ but also in MC density in relation to tumor cell density (% MC of total cells) in CTCL clusters and found a higher MC density in MF (median 2,5%; range 0,45–6,74% MC of total cells) compared to all other CTCL subgroups (see Table 3).

**Table 3:** Mast cell density in relation to tumor cell density in CTCL variants.

Type of lymphoma	Mast cell density (Mast cells/total cells)
	Median (range)
**CTCL**	2,22 (0,15–6,74)
**Mycosis fungoides**	2,53 (0,45–6,74)
**MF IA**	3,54 (0,45–6,74)
**MF IB**	3,65
**MF IIA**	3,03
**MF IIB**	1,72 (0,69–3,29)
**Sezary syndrome**	(0,15;4,05)
**LyP**	1,45 (0,48–2,76)
**SM**	1,10 (0,22–1,58)
**C-ALCL**	0,37

CTCL = primary cutaneous T-cell lymphomas; MF = Mycosis fungoides; LyP = lymphomatoid papulosis; SM = primary cutaneous CD4^+^ small/medium sized pleomorphic T-cell lymphoma C-ALCL = primary cutaneous anaplastic large cell lymphoma;

Regarding MF subgroups, highest MC densities were found in stage I disease (median 3,54%; range 0,45–6,74% MC of total cells) (p = 0.026)

### Degranulation of MC in CTCL

To determine the state of activation of MC we classified MC into 2 groups, degranulated and non degranulated and determined the percentage of each group in all patients and controls. Regarding all CTCL, in the tumor area 41.9% of all MC were degranulated without a significant difference regarding P1 (39,0%; p = 0.368) and P2 (39.8%; p = 0.520). Regarding MF subgroups, a trend towards higher percentages of degranulated MC was found in more advanced stages MF IIA+IIB (median 47,9%; range 28,4–55,8%) than in MF IA+IB (median 37,9%; range 15,0–51,8%; p = 0,100).

In normal skin samples the percentage of degranulated MC was 45.2%. (MC degranulation in CTCL and subtypes see Table 4)

**Table 4:** Percentage of degranulated MC in CTCL variants.

Type of lymphoma		Degranulated MC % median (range)		
	Tumor	P1	P2	Dermis
**CTCL**	41,9 (0–55,8)	39,0 (4,2–57,5)	39,8 (0–70,0)	
**Mycosis fungoides**	42,0 (15,0–55,8)	39,7 (4,2–57,6)	40,0 (7,1–70,0)	
**MF IA**	37,0 (15,0–51,8)	39,4 (4,2–51,6)	37,7 (7,1–66,7)	
**MF IB**	51,0	49,4	45,7	
**MF IIA**	52,4	49,6	50,0	
**MF IIB**	44,0 (28,4–55,8)	38,8 (20,0–57,6)	36,4 (25,0–70,0)	
**Sezary syndrome**	n/a (49,2/0,0)	n/a (12,5/43,3)	n/a (0/48,9)	
**LyP**	38,6 (37,5–48,5)	41,9 (37,9–45,1)	40,0 (38,5–45,4)	
**SM**	42,1 (39,3–43,6)	34,7 (32,1–38,1)	39,6 (23,4–46,3)	
**C-ALCL**	38	34,8	30,0	
**Normal skin**				45,2 (37,1–45,9)

MC = mast cells; CTCL = primary cutaneous T-cell lyphomas; MF = Mycosis fungoides; LyP = lymphomatoid papulosis; SM = primary cutaneous CD4^+^ small/medium sized pleomorphic T-cell lymphoma; C-ALCL = primary cutaneous anaplastic large cell lymhpoma;

## Discussion

In this study we present for the first time a new computer aided image analysis method for MC quantification in immunohistologically stained tissue sections. We applied the method in CTCL to gain more accurate information about presence, number, distribution and state of activation of MC in CTCL variants and clinical stages. We found increased MC counts in the area immediately surrounding CTCL infiltrates and in early stages of MF compared to later stages. With this new computer aided algorithm it was for the first time possible to determine MC density in relation to tumor cell density in CTCL. MC density was found to be increased in MF compared to all other CTCL variants. Regarding the state of activation, around 40% of all MC were degranulated, irrespective of skin condition and relation to CTCL infiltrates.

Up to now there is only one published study about MC in PCL by Rabenhorst et al., where MC numbers were estimated by microscopic counting of 5 high-power fields by 2 independent observers.[32]

The authors of this study found significantly increased numbers of MC in CTCL and CBCL compared to normal skin and concluded on a protumorigenic role of MC in these diseases. This was confirmed in a mouse model of cutaneous lymphoma where tumor growth in MC deficient transgenic mice was significantly decreased.[32]

Similarly, we found higher numbers of MC in CTCL compared to normal skin samples in this study, although the number of control samples was too small (n = 3) to reach sufficient statistical power. However, primary aim of our study was not the interindividual comparison of CTCL with normal skin samples, since due to the high individual and site specific variability of MC counts in health and disease (including CTCL as shown here) sufficient statistical power is hard to achieve. Instead we rather took the approach of an intraindividual comparison between regions with histopathologically evident infiltration of CTCL with adjacent dermal areas, where each case serves as its own control. Doing so, we found a higher MC count in the lymphoma infiltrate and a narrow peripheral region arbitrarily defined by a 30 μm margin compared to more distant dermis. This supports the results of Rabenhorst et al. who found that MC infiltration was particularly prominent at lymphoma rims.[32]

The unique method of MC quantification used in our study enabled us not only to evaluate MC counts relative to area but for the first time also relative to the number of other nucleated cells within the center of the lymphoma infiltrate, which might be of particular pathophysiological relevance due to potential MC—tumor cell interactions. Similar to MC per area we also found a higher relative density of MC in MF compared to all other CTCL subgroups and within MF in the early stage disease subgroup indicating an inverse relationship between the presence of mast cells and clinical stage in CTCL. This seemingly differs from the protumorigenic role of MC as proposed by Rabenhorst et al. and might be due to different technology used and differences in patient series. However, it cannot be excluded that MC in CTCL might have different roles with protumorigenic and anti-tumorigenic functions depending on disease subtype and stage. Another difference to Rabenhorst’s data relates to MC degranulation where automated image analysis, as imployed here, revealed no difference in degranulation rate, whereas conventional microscopy as used by Rabenhorst indicated a significant increase of extensive degranulation in PCL, a difference most likely accounted for by different technology. Looking at the state of activation, it was emphasized that MC activation and following degranulation play an important role in tumor progression. In a review by Marech et al. it was shown in a study on breast cancer, that degranulated MC were mainly present in the peritumoral tissue whereas the non-degranulated MC were in particular located within the tumor tissue.[10,35]

Patruno and Marech et al. studied MC state of activation and microvessel density in a model of canine cutaneous mast cell tumor, and could show a correlation of degranulated MC with a high microvessel density.[36]

Regarding lymphomas, data about MC distribution in different subtypes of lymphomas is sparse. Increased MC number correlated directly with poor prognosis in multiple myeloma and Hodgkin lymphoma.[27,37]

Similar correlations were noted by a Swedish research group, who observed a worse prognosis for a nodular sclerosing subtype of Hodgkin´s lymphoma exhibiting a high MC number.[27]

On the other hand, MC infiltration was shown to be a favorable prognostic factor in diffuse large B-cell lymphoma.[29]

Thus, looking at the literature, in different solid and haematologic human malignancies contrary results about pro- and antitumorigenic MC functions are reported, which show that the role of MC in cancer is still not clear. There is evidence that tissue resident MC have the potential to strongly shape their tissue microenvironment, influencing tumor behavior by participating and regulating inflammatory and immune reactions.[3]

A different signalling environment may influence changes towards or against tumor progression with the mechanisms that underlie these opposing functional outcomes being unclear.[38]

In CTCL the results of our study might indicate an antitumorigenic role of MC. Although the mechanisms of MC interaction with the tumor biology of CTCL remain elusive, in addition to the mechanisms described above, in a T-cell malignancy interactions of MC and malignant and infiltrating lymphocytes might have to be considered. Under certain conditions MC have been described to interact with regulatory T-cells and overcome T-reg mediated immunosuppression, promote the development of effective antitumor immunity and boost the immune response in the tissue.[38]

It might be hypothesized that similar interactions take place in CTCL. Rabenhorst experiments of CTCL-cell stimulation with MC supernatants point to this direction and warrant further investigation in clinically relevant models. The role of MC mediators in tumor growth is very complex and reaches from promoting angiogenesis through VEGF, FGF-2, tryptase and chymase, generating immunosuppression by releasing Il-10, histamine and TNF-alpha, promoting tumor invasiveness by dissolving extracellular matrix through matrix metalloproteases, to promoting inflammation and inhibiting tumor cell growth and tumor cell apoptosis by releasing cytokines such as Interferon-alpha, TNF-alpha, Il-1, Il-4, Il-6.[2]

In CTCL, Rabenhorst et al. provide the only current availaible data about the presence and role of MC mediators. They could show that MC can induce release of IL-6 and additionally a proliferation of CTCL cells in vitro.[32]

Further studies with larger patient cohorts should be performed to clarify whether MC counts are useful as diagnostic or prognostic marker and even may be a future therapeutic target in CTCL.

Considering the limitations of our study, namely (i) a small patient cohort, limiting analysis of prognostic subgroups and (ii) missing information on clinical outcome that could be correlated with MC counts we still consider that the data presented here prove the feasibility of automated image analysis for the study of MC in tissue sections in general and in CTCL in particular.

## Conclusion

This new computer approach not only allows for precise cell identification and quantificaton, but also helps to diminish error from investigator bias inherent to conventional microscopy. By this we introduce a new method for the investigation of MC in the pathology of cancer and other conditions where MC might play a role and at the same time are able to report on novel insights into MC distribution in CTCL. Together with earlier results as described above these data provide an initial basis for further investigation of the role of MC in pathology, disease stage, and prognosis in the various forms of CTCL.

## Supporting Information

S1 Fig Degranulated and non-degranulated mast cells. (TIFF)
