# Supplementary figures and images for: Mast Cells Are Abundant in Primary Cutaneous T-Cell Lymphomas: Results from a Computer-Aided Quantitative Immunohistological Study
Source: PLoS One. 2016 Nov 28;11(11):e0163661. doi: 10.1371/journal.pone.0163661 (PMC5125565; doi:10.1371/journal.pone.0163661)

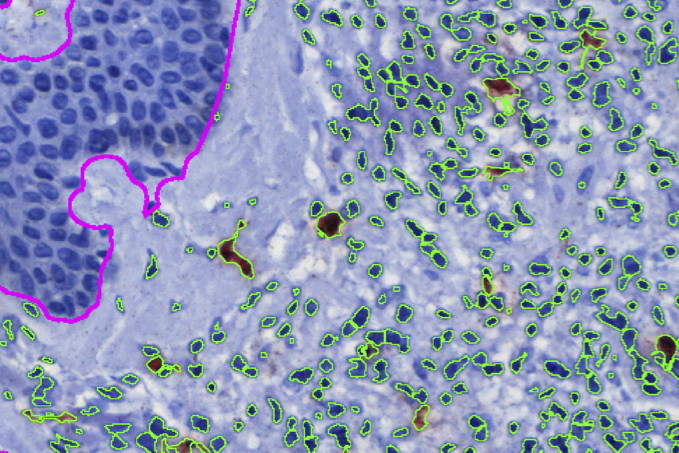

Supplement: S1 Fig — (TIFF) [file pone.0163661.s001.tiff]
